# Genomic Characteristics of a Novel Recombinant Bluetongue Virus Serotype 1 in Yunnan, China

**DOI:** 10.3390/vetsci12090886

**Published:** 2025-09-13

**Authors:** Yunyi Chen, Shimei Luo, Nijing Lei, Zhenghao Ye, Xianping Ma, Shaoyu Yang, Huaijie Jia, Guangyu Qi, Guanghua Wang, Huashan Yi

**Affiliations:** 1 College of Veterinary Medicine, Southwest University, Rongchang, Chongqing 402460, China; 2 Guangxi Key Laboratory of Marine Environmental Disaster Processes and Ecological Protection Technology, Beibu Gulf University, Qinzhou 535011, China; 3 State Key Laboratory for Animal Disease Control and Prevention, Lanzhou Veterinary Research Institute, Chinese Academy of Agricultural Sciences, Lanzhou 730046, China; 4 Huapai Biotechnology (Grounp) Co., Ltd., Chengdu 641400, China; 5 Qinghai Academy of Animal Sciences and Veterinary Medicine, Qinghai University, Xining 810016, China

**Keywords:** bluetongue virus, genome-wide reassortment, NGS, phylogenetic analysis, evolutionary analysis

## Abstract

Bluetongue virus belongs to the Reoviridae family, which causes morbidity in ruminants. Herein, we report the full-length genome sequence of a recombinant BTV strain of serotype 1 (YNDH/103/2013) isolated from sentinel cattle in Yunnan Province, China, in 2013. To identify and understand the characteristics of YNDH/103/2013, we screened closely related viruses using Simplot 3.5.1 software and conducted verification via phylogenetic analysis using MEGA 11.0.13 software. Phylogenetic analysis and segment examination showed that the YNDH/103/2013 strain likely originated from multiple recombination events involving prevalent strains such as Y863, NRT37/ABT/HSR, and YTS-4 in China and India. The genome of BTV-1/YNDH/103/2013 derives from at least three reassortments with these strains, showing recombination breakpoints mainly in segments 4, 6, 8, and 9. This study improves our understanding of the origin and spread of BTV-1 in China.

## Simple Summary

This study presents the full genome sequence of a recombinant BTV-1 strain (YNDH/103/2013) isolated from sentinel cattle in Yunnan, China, in 2013. Phylogenetic and recombination analyses of all 10 genome segments show that YNDH/103/2013 likely originated from multiple recombination events involving field strains, such as Y863, NRT37/ABT/HSR, and YTS-4, with recombination breakpoints mainly in genome segments 4, 6, 8, and 9. These findings reveals the complex evolution of BTV-1, highlighting how genetic recombination and reassortment drive to viral diversity. This study enhances our understanding of BTV-1’s epidemiology and genetic variation, providing critical insights for outbreak surveillance and the development of effective control strategies in livestock.

## 1. Introduction

Bluetongue virus (BTV) is the etiological agent of bluetongue disease (BT), which affects both domestic and wild ruminants, such as white-tailed deer, and is transmitted between mammalian hosts almost exclusively by biting midges [1,2]. BTV belongs to the Orbivirus genus of the Reoviridae family, and its genome comprises 10 segments (Seg-1-Seg-10) of double-stranded RNA (dsRNA) encoding seven structural proteins (VP1-VP7) and five nonstructural proteins (NS1, NS2, NS3/NS3a, NS4 and NS5) [3,4]. After infection, a range of symptoms, including fever, nasal discharge, leukopenia, mucosal congestion, inflammation, and lobular pneumonia-like lesions, may occur [3,5].

To date, 36 distinct BTV serotypes (BTV-1 to BTV-24 and atypical serotypes BTV-25 to BTV-29) have been found, and several serotype-unassigned BTV strains have been isolated [6,7,8,9]. Over the past four decades, BT has been endemic in China, and BTV strains belonging to eleven different serotypes (BTV-1, BTV-2, BTV-3, BTV-4, BTV-9, BTV-12, BTV-15, BTV-16, BTV-21, BTV-24, and BTV-29) have been isolated from southeastern, southwestern, and northwestern China, specifically Yunnan, Guangdong, Jiangsu, Hubei, Guangxi and Xinjiang Provinces [7,10,11,12].

Genetic diversity among BTV strains can be attributed to heterologous or homologous serotype virus reassortment and mutation [13,14]. Recent next-generation sequencing (NGS)-based studies have uncovered increasing evidence of complex reassortment patterns in Southeast Asian BTV strains, highlighting the region as a hotspot for viral evolution [15,16]. China’s Yunnan Province, which borders Myanmar, Laos, and Vietnam, is a critical geographical junction where multiple BTV lineages from different regions might converge, creating unique opportunities for genetic exchange. Reassortment is a genetic process that occurs when two or more virus strains co-infect a single host cell and exchange their gene segments to generate virus progeny with a new genotype that is a hybrid of the two infecting genotypes [13,15].

Despite the documented circulation of multiple BTV serotypes in Yunnan Province, the detailed genomic characteristics and evolutionary relationships of contemporary BTV-1 isolates from this region remain poorly understood. The YNDH/103/2013 strain presents a unique opportunity for researchers to investigate BTV evolution in this geographical hotspot, as preliminary analysis has indicated that this strain has unusual replication characteristics in cell culture, warranting comprehensive genomic investigation. We hypothesize that the YNDH/103/2013 isolate represents a novel reassortant strain, resulting from genetic exchange between multiple BTV lineages circulating in the China-Southeast Asia border region. The objectives of this study were to: (1) determine the complete genome sequence and phylogenetic relationships of BTV-1/YNDH/103/2013; (2) identify potential reassortment events and parental lineages using computational approaches; and (3) provide insights into BTV evolution and circulation patterns in southwestern China.

## 2. Materials and Methods

### 2.1. Ethical Considerations

All procedures involving viral isolation and cell culture work were conducted in accordance with institutional biosafety guidelines. The study used archived viral samples from routine surveillance activities and did not involve additional animal experiments.

### 2.2. Strains and Cells

The YNDH/103/2013 strain was isolated from sentinel cattle in Dehong City, Yunnan Province, China, in July 2013. It has been preserved at −80 °C under strict biosafety protocols. Baby hamster kidney (BHK-21) cells were purchased from the American Type Culture Collection (Manassas, VA, USA). Primary ovine pulmonary microvascular endothelial cells (OLMECs) were purchased from iCell Cybiocon (Shanghai, China).

### 2.3. Experimental Infection of BHK-21 Cells and OLMECs with BTV-1/YNDH/103/2013

BHK-21 cells and OLMECs were resuscitated in a water bath at 37 °C from −80 °C storage in our laboratory and centrifuged at 1000 rpm for 5 min. The cells were resuspended in 5% fetal bovine serum (Gibco, Carlsbad, CA, USA), 1% triple antibody (streptomycin–penicillin–amphotericin B), and 90% MEM or 5% fetal bovine serum, 1% double antibody (streptomycin–penicillin), 1% endothelial cell nutrition factor, and 90% endothelial cell special medium, evenly spread in a T25 cell culture bottle and cultured at 37 °C in a 5% CO_2_ cell incubator. When the cell density exceeded 85%, the cells were passaged and re-plated in 24-well cell culture plates. The BTV-1 strain was added to the 24-well cell culture plate, and the viral titer was determined via plaque formation assay, estimated as plaque-forming units per milliliter (PFU/mL). BTV-1/YNDH/103/2013 was inoculated into BHK cells at an MOI of 1, and the cultures were harvested upon complete cytopathic effect (CPE).

### 2.4. Preparation and Detection of Viral Genomic dsRNA

Collect the cell samples obtained following post-infection procedures outlined in Section 2.3, and centrifuge them at a low speed (2000 rpm, 10 min). After centrifugation, the supernatant was discarded. Next, 3 mL of TRIzol™ reagent (Invitrogen, Carlsbad, CA, USA) was added to the cell mixture, and the sample was thoroughly lysed. Subsequently, 0.6 mL of chloroform (Sigma-Aldrich, Taufkirchen, Germany) were added, followed by vigorous mixing and incubation at room temperature for 2–3 min. Following centrifugation, the upper aqueous phase was collected, and an equal volume of isopropanol (Sigma-Aldrich, Taufkirchen, Germany) was added. The mixture was allowed to stand, then centrifuged again, and the supernatant was removed to obtain the RNA pellet. Finally, the RNA pellet was washed with 75% ethanol and dissolved in 50 μL of UltraPure™ DEPC-Treated Water (Invitrogen, California, USA). The extracted total RNA was added to 1 μL of nuclease S1 and incubated at 37 °C for 30 min. Then, the nucleic acid was purified using the Monarch RNA Cleanup Kit (New England Biolabs, Ipswich, MA, USA) according to the instructions. The dsRNA of 5 μL of the purified viral genome was obtained, and the migration pattern of the dsRNA was detected via 1% agarose gel electrophoresis (AGE).

### 2.5. Library Construction, and Next-Generation Sequencing

RNA was extracted from samples of clarified infected cell lysates using the TRIzol method, as detailed in Section 2.4, and prepared for next-generation sequencing (NGS). To exclude potential contamination or coinfection, RNA samples were treated with DNase I and quality-checked using an Agilent 2100 Bioanalyzer. Illumina sequencing and library construction were performed at Shanghai Tanpu Biotechnology Co., Ltd. (Shanghai, China). In brief, the NEBNext^®^ Ultra™ II RNA Library Prep Kit (NEB, Ipswich, MA, USA) was used for library construction. After adapter ligation, 10 cycles of PCR amplification were performed to enrich the sequencing target. The libraries were pooled in equimolar amounts, denatured, and diluted to the optimal concentration before sequencing. The Illumina NovaSeq 6000 System (San Diego, CA, USA) was used to sequence the 150 bp pair-end reads of the genome.

### 2.6. Genome Sequence, Assembly, Annotation, and Phylogenetic Tree Construction

Read quality trimming was performed via fastp (OpenGene/fastp. https://github.com/OpenGene/fastp (accessed on 20 September 2024)) to remove sequencing adapters and low-quality reads, including those scoring <Q20. Ribosomal RNA and host read subtraction by read mapping was performed with the BBMAP (BioInfoTools/BBMap. https://github.com/BioInfoTools/BBMap (accessed on 25 September 2024)) program. De novo genome assembly was performed via SPAdes v3.14.1 (ablab/spades. https://github.com/ablab/spades (accessed on 2 October 2024)) [17,18]. These extracted assembled scaffolds limited the minimum contig length to 100 bases to obtain the best BLAST (http://blast.ncbi.nlm.nih.gov/Blast.cgi (accessed on 10 October 2024))hits with the NCBI nt database [19]. Pairwise distance (nt/aa) calculations and phylogenetic tree construction were conducted via MEGA 11.0.13 software [20], with maximum likelihood (ML) or neighbor-joining (NJ) methods tested by bootstrapping 1000 replicates. Where appropriate, the nomenclature for the geographically based phylogenetic groups (topotypes) of BTV segments is provided as described by Maan et al. [21].

### 2.7. Analysis of Recombination Events in the Complete Genome

Potential reassortment or recombination events in the complete genome of strain BTV-1/YNDH/103/2013 were identified using the Recombination Detection Programme (RDP 4 Version 4.33) through different algorithms (RDP, GENE-CONV, Bootscan, MaxChi, Chimaera, SiScan, and 3Seq) [22]. Default parameters were used with a significance threshold of *p* < 0.05, and events detected by at least three methods were considered significant. The potential reassortment or recombination events were further verified via similarity plot (SimPlots) analysis in SimPlot version 3.5.1 [23] using a sliding window of 200 bp with a step size of 20 bp, and finally, a phylogenetic tree was constructed using MEGA 11.0.13 software.

## 3. Results

### 3.1. Identification of the dsRNA Genome Structure and NGS Analysis

**Figure 1:**
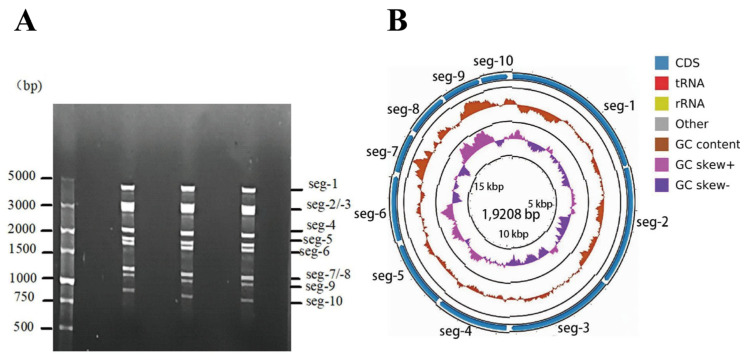
dsRNA genome identification and analysis. (**A**) Electrophoresis profile of the dsRNAs; (**B**) genome map. Note: CDS: the cds fragment after assembly sequence annotation; GC content: displays the change in GC content in the assembly sequence (calculated using a sliding window with different lengths according to the sequence length; for contig length < 10,000, the sliding window length is 50, and for contig length < 100,000, the sliding window length is 500); GCskew ±: GC content offset, where GCskew = (G − C)/(G + C), used to measure the relative content of G and C. If G is greater than C, the value of GCskew is positive, and if G is less than C, it is negative.

The viral genomic dsRNA detection results demonstrate that the genome consists of ten segments exhibiting a 3-3-3-1 migration pattern (Figure 1A). Genome PCR product sequencing produced 8,237,478 raw reads. Following quality filtering, 7,661,078 reads were retained, resulting in a data retention rate of 92.99%. Sequence assembly based on k-mer analysis demonstrated that 99.98% of the high-quality reads were assembled into 10 contigs, with an N50 length of 903 bp. Sequencing depth analysis revealed that 86.12% of the contigs exhibited an average sequencing depth exceeding 100×. This indicates that the majority of genomic regions are adequately covered, thereby providing a baseline for accurate variant detection and reliable sequence analysis. The complete genome map of the BTV-1/YNDH/103/2013 strain was constructed after achieving 99.95% genome coverage through sequence assembly and splicing (Figure 1B). The whole genome comprises 19,208 bases, and genome Seg-1–Seg-10 of YNDH/103/2013 are 3940, 2958, 2773, 1986, 1777, 1636, 1146, 1125, 1052 and 815 bp, respectively. The complete CDSs of Seg-1 to Seg-10 encoded 3909 (VP1), 2886 (VP2), 2706 (VP3), 1854 (VP4), 1659 (NS1), 1581 (VP5), 981 (VP7), 1065 (NS2), 993 (VP6, NS4), and 690 (NS3/NS3a) nucleotides, respectively. The full genome sequences (Seg-1 to Seg-10) have been deposited in GenBank (accession numbers: PQ168272–PQ168281).

### 3.2. Phylogenetic Analysis of BTV-1/YNDH/103/2013

**Figure 2:**
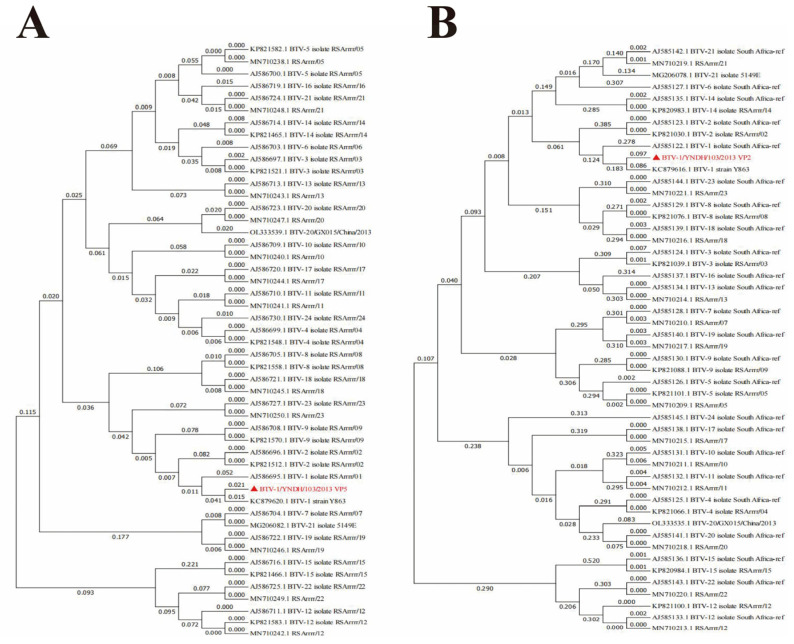
Phylogenetic Analysis of BTV-1/YNDH/103/2013. (**A**) Analysis of Segment 2 (S2); (**B**) Analysis of Segment 6 (S6). Phylogenetic trees were constructed using MEGA 7.0. The numbers at each branch indicate branch distances. YNDH/103/2013 is depicted with a red triangle. RSArrrr/01-24 represents the BTV-1–24 serotype reference strains deposited in GenBank.

**Figure 3:**
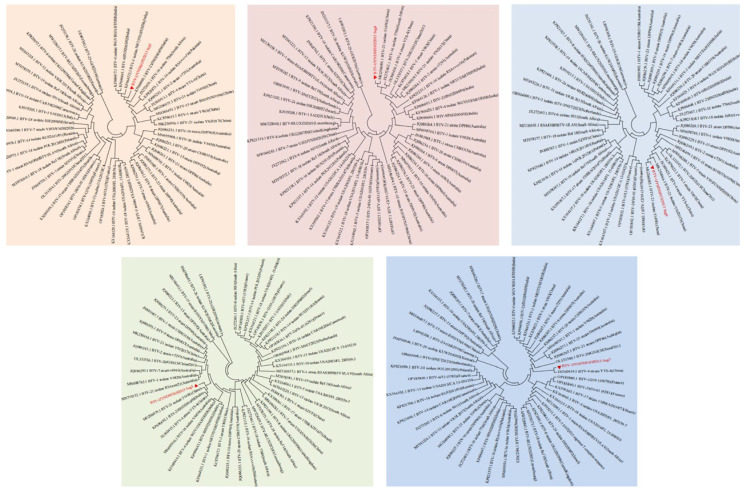
Phylogenetic trees constructed based on the VP1 (Seg-1), VP4 (Seg-4), VP6 (Seg-9), VP3 (Seg-3), and VP7 (Seg-7) gene sequences of BTV-1/YNDH/103/2013. The neighbor-joining method was employed using MEGA 7.0 software. The viral genes analyzed in this study are indicated by solid red triangles.

**Figure 4:**
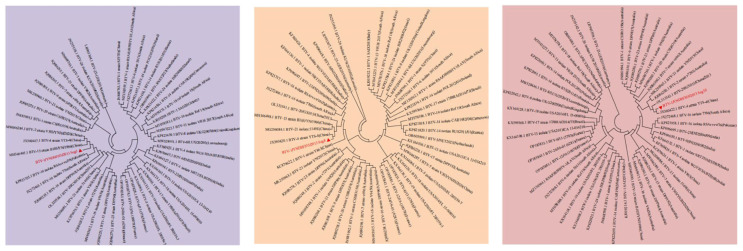
Phylogenetic trees based on the NS1 (Seg-5), NS2 (Seg-8), and NS3/NS3a (Seg-10) gene segments of BTV-1/YNDH/103/2013. The neighbor-joining method was applied using MEGA 7.0. The viral genes studied in this study are marked by solid red triangles.

The Seg-2 and Seg-6 sequences of BTV-1 from the reference strains and strains isolated from different countries available in GenBank were aligned with the sequences obtained in our study. Phylogenetic analysis of the full-length genomes of Seg-2 and Seg-6 revealed that BTV-1/YNDH/103/2013 clustered with BTV-1 Y863 and presented high nucleotide sequence identity (≥95%) with BTV-1, confirming that the isolate BTV-1/YNDH/103/2013 belonged to BTV serotype 1 (Figure 2). Furthermore, BTV-1/YNDH/103/2013, BTV-1Y863, and BTV-4 YTS-4, isolated in Yunnan, clustered together, indicating a distinctive Chinese lineage (Figure 3 and Figure 4). Similar phylogenetic relationships were observed when the conserved genomic segments (Seg-1, Seg-3, Seg-4, Seg-5, Seg-7, Seg-8, Seg-9, and Seg-10) were analyzed separately.

### 3.3. Reassortment Analysis of BTV-1/YNDH/103/2013

**Figure 5:**
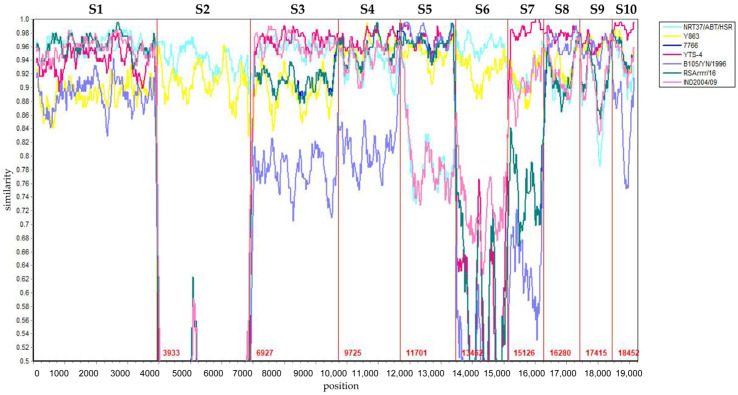
Simplot analysis of eight BTV isolates. BTV-1/YNDH/103/2013 was used as the query. The cyan, yellow, blue, aubergine, blue-purple, green, and pink lines represent the strains NRT37/ABT/HSR, Y863, 7766, YTS-4, B105/YN/1996, RSArrr/16, and IND2004/09, respectively. The Y-axis indicates the degree of sequence similarity, and the X-axis indicates the genomic positions of segments S1 to S10.

**Figure 6:**
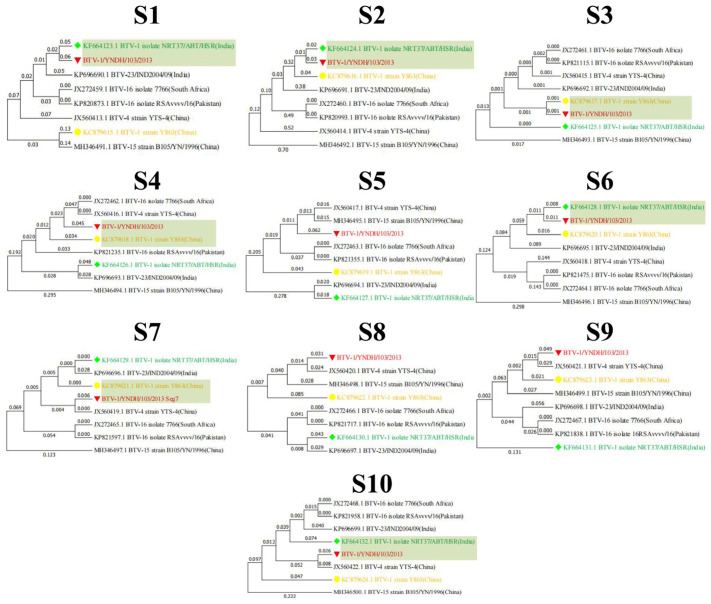
Maximum likelihood phylogenetic trees of the Seg-1(S1)–Seg-10(S10) of BTV and related recombinant strains. The numbers on the branch represent evolutionary distances.

Reassortment analysis was performed using Simplot 3.5.1 software. Computational analysis indicated that the YNDH/103/2013 isolate potentially originated from multiple reassortment events involving field strains, such as Y863, NRT37/ABT/HSR, and YTS-4, widely circulating in the Yunnan region of China. YNDH/103/2013 exhibits multiple reassortment breakpoints localized at Seg-4, -6, -8, and Seg-9 of BTV (Figure 5). The phylogenetic analysis of individual segments supported these findings, with Seg-1, -2, and Seg-6 showing highest similarity to NRT37/ABT/HSR, whereas Seg-4, -7, -9, and Seg-10 presented high homology with YTS-4. Moreover, Seg-5 strongly resembled RSArrr/16 (Figure 6).

RDP4.0 analysis confirmed potential reassortment events in the BTV-1/YNDH/103/2013 isolate strain (Table 1). Four significant reassortment events were detected, with the BTV-1/YNDH/103/2013 isolate predominantly deriving from the BTV-1 strain NRT37/ABT/HSR, with contributions from the BTV-1 strain Y863. The primary reassortment breakpoint was located between nucleotides 10,465 and 13,413 (BTV Seg-5).

## 4. Discussion

The serotype of BTV is primarily determined by the VP2 and VP5 proteins [24,25]. Phylogenetic analysis based on the S2/VP2 and S6/VP5 sequences revealed that the YNDH/103/2013 strain exhibits the highest sequence similarity to the BTV-1 strain Y863 (GenBank accession number: KC879624.1). BLASTN analysis of other genomic segments of YNDH/103/2013 revealed that Seg-3, -7, -8, and -10 all presented high nucleotide sequence identity (≥97.04%) with the Chinese BTV-4 isolate (YTS-4). Seg-1 exhibited the highest degree of similarity to the corresponding fragments of the Indian BTV-1 type. Seg-5 and Seg-9 of YNDH/103/2013 exhibited high similarity to the Chinese BTV-21 strain, isolated in 2013 and 2017, with nucleotide identity values of 99.14% and 96.96%, respectively. These findings suggest that genetic reassortment or recombination may occur among different BTV serotype strains, potentially giving rise to novel BTV lineages.

Our study suggests that the BTV-1/YNDH/103/2013 strain may be a genetically reassorted virus, with segments potentially deriving from BTV-1 Y863, BTV-4 YTS-4, NRT37/ABT/HSR and RSArrrr/16. Notably, in the year following the discovery of YNDH/103/2013 (2014), Yang et al. reported the complete genomic sequence of the BTV-7 strain GDST008 isolated in China. Comparative analysis revealed that this strain shares a close genetic relationship with the BTV-1 strain Y863 (isolated in China in 1979) and the BTV-4 strain YTS-4 (isolated in 1996) [26]. These findings suggest the presence of a long-standing, continuously circulating BTV lineage in Yunnan Province that has undergone genetic exchange with other foreign strains at different time points. Previous studies have elucidated that gene mutations and segment rearrangements are pivotal evolutionary mechanisms propelling the diversification of BTV. As RNA viruses, BTV possess the capability to undergo genetic recombination at diverse levels, both within and across genera. This recombination potentially not only expands their host range but also significantly enhances their ability to evade host immune responses [27,28,29].

The BTV-1 strain Y863 was initially isolated from a herd of livestock that exhibited severe clinical symptoms of BT during an outbreak in Shizhong County, Yunnan Province [10]. In contrast, the YNDH/103/2013 strain was isolated from a sentinel cow that displayed no clinical symptoms during routine monitoring. This discrepancy suggests that the newly generated recombinant virus strain may exhibit phenotypic characteristics that are distinct from those of the parental virus, which could potentially result in altered transmission dynamics or pathogenicity. NRT37/ABT/HSR and RSArrrr/16 strains both originated from India. Given that India is an endemic region for multiple BTV serotypes, it inevitably provides numerous opportunities for genomic segment recombination [21]. RSArrrr/16 exhibits 99% sequence identity to the Chinese BTV-16 strain BN96/16, which was isolated from sheep in Yunnan in 1996 [30]. This high degree of sequence similarity suggests that gene rearrangement or recombination events between Indian and Chinese BTV strains may have already occurred as early as the 1990s.These findings not only highlight the extensive geographical distribution and efficient transmissibility of BTV, but also reinforce cross-border collaborative research to underlying genetic recombination and evolutionary dynamics, as well as for prevention and control strategies of BT.

This study has several limitations. First, the reassortment events were inferred solely through computational analysis without experimental validation. Second, the pathogenicity and replication characteristics of the YNDH/103/2013 strain in natural hosts remain undetermined, as no corresponding animal experiments were conducted. Third, the limited availability of complete genome sequences in public databases might have affected the accuracy of reassortment analysis, particularly for Seg-5, where unknown recombinant parents were encountered. Future studies should focus on experimental validation of the proposed reassortment events through reverse genetics approaches. Additionally, animal pathogenicity studies can help determine the biological significance of the observed genetic changes. Expanded genomic surveillance in the China–Southeast Asia border region would provide better understanding of BTV circulation patterns and evolution.

## 5. Conclusions

This study provides the genomic characterization of a BTV-1 isolate from Yunnan Province, China, with evidence suggesting complex reassortment events involving multiple parental lineages. The computational analysis indicates that BTV-1/YNDH/103/2013 may represent a novel reassortant strain derived from at least three different BTV lineages circulating in China and India. These findings contribute to our understanding of BTV evolution and highlight the importance of continued genomic surveillance in regions where multiple BTV serotypes co-circulate. However, experimental validation and pathogenicity studies are needed to confirm the biological significance of the proposed reassortment events.

## Figures and Tables

**Table 1:** Recombination of the whole genome of BTV-1/YNDH/103/2013.

Number of Recombinant Events	Recombinant Sequences	Major Parents	Minor Parents	Breakpoint Position	Detection Methods						
					R	G	B	M	C	S	T
1	YNDH/103/2013	NRT37/ABT/HSR	Y863	10,465–13,413 nt	+	+	+	+	+	+	+
2	YNDH/103/2013	NRT37/ABT/HSR	RSArrrr/16	11,376–13,312 nt	+	+	+	+	+	+	+
3	YNDH/103/2013	NRT37/ABT/HSR	YTS-4	15,117–16,277 nt	+	-	+	+	+	+	+
4	YNDH/103/2013	NRT37/ABT/HSR	YTS-4	18,511–19,080 nt	+	+	+	+	+	+	+

Note: R, RDP; G, GENECONV; B, Bootscan; M, Maxchi; C, Chimera; S, Siscan; T, Phylpro.

## Data Availability

The datasets used and/or analyzed during the current study are available. Sequence data that support the findings of this study have been deposited in the National Center for Biotechnology Information under the following GenBank accession numbers: PQ168272–PQ168281.
